# Inhibition or Reversal of the Epithelial-Mesenchymal Transition in Gastric Cancer: Pharmacological Approaches

**DOI:** 10.3390/ijms22010277

**Published:** 2020-12-29

**Authors:** Joanna Kozak, Alicja Forma, Marcin Czeczelewski, Paweł Kozyra, Ryszard Sitarz, Elżbieta Radzikowska-Büchner, Monika Sitarz, Jacek Baj

**Affiliations:** 1 Department of Human Anatomy, Medical University of Lublin, 20-090 Lublin, Poland; joanna.kozak@umlub.pl; 2 Department of Forensic Medicine, Medical University of Lublin, 20-090 Lublin, Poland; aforma@onet.pl (A.F.); marcin.czeczelewski@gmail.com (M.C.); 3 Student Research Group, Independent Radiopharmacy Unit, Faculty of Pharmacy, Medical University of Lublin, PL-20093 Lublin, Poland; pawekoz@interia.pl; 4 1st Department of Psychiatry, Psychotherapy and Early Intervention, Medical University of Lublin, Gluska Street 1, 20-439 Lublin, Poland; e.sitarz@hotmail.com; 5 Department of Plastic Surgery, Central Clinical Hospital of the Ministry of the Interior in Warsaw, 01-211 Warsaw, Poland; elzbieta.radzikowska@gmail.com; 6 Department of Conservative Dentistry with Endodontics, Medical University of Lublin, 20-090 Lublin, Poland; mksitarz@gmail.com

**Keywords:** gastric cancer, epithelial-mesenchymal transition, EMT, pharmacotherapy

## Abstract

Epithelial-mesenchymal transition (EMT) constitutes one of the hallmarks of carcinogenesis consisting in the re-differentiation of the epithelial cells into mesenchymal ones changing the cellular phenotype into a malignant one. EMT has been shown to play a role in the malignant transformation and while occurring in the tumor microenvironment, it significantly affects the aggressiveness of gastric cancer, among others. Importantly, after EMT occurs, gastric cancer patients are more susceptible to the induction of resistance to various therapeutic agents, worsening the clinical outcome of patients. Therefore, there is an urgent need to search for the newest pharmacological agents targeting EMT to prevent further progression of gastric carcinogenesis and potential metastases. Therapies targeted at EMT might be combined with other currently available treatment modalities, which seems to be an effective strategy to treat gastric cancer patients. In this review, we have summarized recent advances in gastric cancer treatment in terms of targeting EMT specifically, such as the administration of polyphenols, resveratrol, tangeretin, luteolin, genistein, proton pump inhibitors, terpenes, other plant extracts, or inorganic compounds.

## 1. Introduction

Gastric cancer (GC) attributes for 8.3% of all cancer deaths and is the third leading cause of cancer-related deaths worldwide [1,2,3]. Annually, about 990,000 patients are diagnosed with GC, of whom 75% die, mainly because of the lack of enough sensitive and specific biomarkers that would provide early detection of this malignancy [4,5]. GC is approximately two times more prevalent among males compared to females [6]. Except for the prevalence of GC, the differences between sexes are also observed as different survival rates or clinicopathological features; generally, females present better cancer-specific survival and overall survival rates compared to males [7,8]. Besides, the early and undifferentiated GC is much more frequently observed in female GC patients. In terms of distribution in the world, GC is the most prevalent in Central and South America, East Asia, and Eastern Europe [9]. The incidence rate of GC showed a decreasing trend in recent years [10]. The advances in the development of detection and treatment strategies have contributed to the five-year survival rate of approximately 60% in Japan [11]; however, the worldwide average five-year survival rate remains at 40% [12].

GC is a disease of a multifactorial etiology that is induced by numerous environmental and genetic factors [13,14]. The recognized risk factors of GC include the family history, diet, alcohol consumption [15,16], smoking, or Epstein-Barr Virus (EBV) infection [1], as well as a prolonged intake of non-steroidal anti-inflammatory drugs (NSAIDs) or proton pump inhibitors (PPIs) [17,18]. So far, *Helicobacter pylori* (*H. pylori*) constitutes the major cause of GC [19,20].

Treatment strategies of GC differ depending on the clinical course, the severity, as well as either the presence or lack of the metastasis. Currently, the standard treatment strategy of GC is the radical (total or subtotal) gastrectomy with D2 lymphadenectomy. Endoscopic mucosal resection is preferred when there is no metastasis to the lymph nodes; other common strategies include perioperative chemotherapy or a combination of the chemotherapy with radiotherapy [21]. Current standards of GC treatment also include the targeted therapies of most commonly mutated genes or proteins [22,23,24]. Therefore, specific processes constituting the hallmarks of GC such as angiogenesis or epithelial-mesenchymal transition (EMT) can be inhibited or even reversed improving the clinical outcome of GC patients.

## 2. Epithelial-Mesenchymal Transition in Gastric Carcinogenesis

Epithelial-mesenchymal transition (EMT) is the process of re-differentiation of the epithelial cells into the mesenchymal ones; the reversal of EMT is called mesenchymal-epithelial transition (MET). Physiologically, EMT is observed during organogenesis (type 1 EMT), development and remodeling of tissues, as well as the regeneration of wounds (type 2 EMT). Apart from the physiological functions, EMT constitutes one of the most crucial hallmarks of carcinogenesis (type 3 EMT). Crucial aspects of EMT include destabilization and disorganization of the adherens junctions, desmosomes, and claudins, which further impairs the epithelial junctions promoting cellular transition into the mesenchymal phenotype [25]. The above-mentioned process is due to the switch of the type 1 cadherin (E-cadherin) into the neural cadherin (N-cadherin) that is promoted by the deregulations in the epithelial gene expression and activation of the genes responsible for the induction of the mesenchymal phenotype [26]. Besides, crucial events of EMT also include the loss of cellular polarity and reorganization of the cellular cytoskeleton structure; EMT also facilitates the induction of angiogenesis [27,28]. Cells that have undergone the EMT process are resistant to apoptosis and present enhanced motility [29]. Except for stimulating the invasiveness properties, EMT contributes to tumor metastasis and heterogeneity; it might also facilitate the resistance to cell death, leading to multi-drug resistance [30]. EMT promotes the induction of the cancer stem cell (CSC) phenotype, which facilitates the progression of GC as well as further cellular stemness [31]. Both in vitro and in vivo studies showed that there is an interplay between EMT and inflammation since EMT facilitates the secondary release of pro-inflammatory cytokines; besides, chronic inflammation occurring in the GC microenvironment also triggers EMT [32,33,34,35]. Similarly, EMT and reactive oxygen species (ROS) formation are also associated with one another.

Epithelial markers of which expression is decreased during EMT include the E-cadherin, claudin, occludin, cytokeratins, desmoglein, laminin-1, zona-occludens 1 (ZO-1), or Syndecan. Contrarily, the expression of the mesenchymal markers is significantly increased and includes the N-cadherin, vimentin, Snail, Slug, fibronectin, OB-cadherin, and β-catenin, among others [36]. Alterations in the expression of EMT-associated markers, including a decrease in E-cadherin and an increase in N-cadherin, are closely associated to the invasive and metastatic capacity of cancer cells. The upregulation of the EMT-related genes is mainly due to the mutations in the WNT5A and p53 genes [37]. The overexpression of EMT-related proteins including vimentin and TWIST1 and decreased expression of E-cadherin with programmed cell death factor 4 (PDCD4) are associated with a malignant degree of GC patients [38]. Moreover, the upregulation of the abovementioned mesenchymal markers along with downregulation of epithelial markers lead to the progression of cellular migration, invasion, and proliferation [39]. EMT transcription factors are crucial in the induction of the resistance against numerous cancer therapies; the presence of EMT in the GC microenvironment is generally associated with poorer clinical outcome primarily because of the acquisition of the resistance to most anti-cancer treatment therapies [40,41]. Besides, EMT constitutes a crucial regulator of phenotypic plasticity in cancer cells as well as induces the suppression of anoikis [42].

The following manuscript is primarily based on the review of the experiments performed on a single cell line or cell lines frequently used in GC research such as SGC-7901. The undifferentiated SGC-7901 cell line was originally established from the surgically resected metastatic lymph node of a female patient. SGC-7901 cell line is histologically consistent with stage 4 GC with remarkable peritoneal invasion [43]. Furthermore, the human GC cell line SGC-7901 is characterized as highly tumorigenic and metastatic and for these reasons is relevant to the EMT study [44]. In our opinion, the experiments performed on human cell lines using standardized and proven methodology provide replicable and valuable results. Moreover, GC cell lines are widely accepted models to investigate the biological characteristics and molecular mechanisms of an experimental therapy for gastric tumors. Only the verifiable and replicable study results make a promising step toward new therapeutics development. These preliminary results are frequently taken into consideration before the in vivo experiments. This approach allows for the funds restriction and minimizes the suffering of experimental animals.

## 3. Generation of the Reactive Oxygen Species

Reactive oxygen species (ROS) are naturally produced by cells through the aerobic metabolic processes [45]. The major sources of cellular-derived ROS are mitochondria and mitochondrial electron transport system (ETS) [46]. Additionally, the exogenous factors such as ionizing radiation, UV light, or some drugs contribute to the overproduction of ROS [45]. Mitochondrial ROS appears as some electrons escape the ETS and bind to oxygen to form superoxide radicals [46,47]. Thereafter, the superoxide dismutase (SOD) converted superoxide radicals into hydrogen peroxide. In the presence of reduced iron or copper, hydrogen peroxide can be converted into highly toxic hydroxyl radicals [47,48]. Another source of cellular-derived ROS is the hepatic endoplasmic reticulum (microsomes) with a cytochrome 450 monooxygenase system that catalyzes the oxidation of NADPH, leading to the production of superoxide [46,47]. ROS has the potency to induce oxidative stress in living cells through interaction with DNA, lipids, and protein molecules leading to DNA damage, mutation, lipids oxidation, and protein dysfunction [49]. However, healthy cells neutralize ROS by activating antioxidant systems that consist of enzymatic antioxidants such as SOD, thioredoxin (Trx), catalase (CAT), and glutathione peroxidases (GPxs). Moreover, ROS neutralizing potency has also some proteins that do not exhibit direct catalytic activity toward ROS such as Sestrins 1 to 3 (SESN1, SESN 2, and SESN 3) [50]. It seems that oxidative stress is due to an imbalance between ROS production and detoxification resulting in abnormal accumulation of ROS in cells [51]. Excessive and sustained ROS production leads to dysregulation of pivotal cellular processes including growth, proliferation, differentiation, migration, and apoptosis [45,47,48]. Persistent DNA damage by the elevated ROS levels could cause replication errors, genomic instability, activation of oncogenes, and inactivation of tumor suppressor genes, and ultimately induce the development of cancer [52,53,54,55,56]. It is worth noting that oxidative stress and ROS are not sufficient to explain the onset and development of all kinds of cancers. However, we cannot completely ignore the findings that ROS and tumor biology are closely connected.

## 4. Interplay between ROS and EMT in Gastric Cancer

Importantly, ROS generation is involved in the EMT process by the following activities: Cytoskeleton remodeling (e.g., by actin and tubulin regulation), regulation of extracellular matrix (ECM) remodeling (e.g., by integrin modification), cell–cell junctions’ regulation (e.g., by the interaction with nuclear factor kappa B (NF-κB) and hypoxia-inducible factor (HIF-1α)), and regulation of cell motility (e.g., by the regulation of proto-oncogene tyrosine-protein kinase Src (Scr) and focal adhesion kinase (FAK)) [47,57,58]. In this section, we will highlight recent progress in understanding the molecular basis of ROS-regulated EMT in GC cells and discuss potential implications in therapeutic strategy.

Cytoskeleton remodeling is an important step for cell migration due to the dynamic alteration of cellular protrusions that occurs [58]. Recent studies have shown that actin remodeling in GC cells is attributed to increasing ROS levels. Cai et al. speculated that ras homolog gene family member A (RhoA), a critical signaling molecule regulating a variety of cellular processes such as cytoskeletal organization, adhesion, and apoptosis, is considered responsive to ROS and redox state [59]. Via a functional analysis, they demonstrated that oxidative stress caused by emodin, a ROS producer, in combination with arsenic trioxide (ATO) led to RhoA inactivation that resulted in structural disruption of focal adhesion complex resulting in anoikis [59]. Focal adhesion sites are specific areas on the cell membrane where cells attach to ECM. They are complexes of structural and signaling proteins, anchoring actin filaments, and microtubules to the plasma membrane where integrins locate [60]. Even though the research was on RhoA function in anoikis resistance of GC cells, we would like to stress that RhoA is a signaling mediator of the actin cytoskeleton remodeling. Therefore, the status of oxidative stress might be a therapeutic strategy for the inhibition of RhoA in cancer cells and indirectly influence cancer cell EMT. Similarly, the importance of RhoA in cell migration and invasion in GC cells was also demonstrated by Murray et al. It was demonstrated in a functional study that neuroepithelial cell transforming gene 1 (NET1) is upregulated in GC cells and participate in proliferation and invasion. Further, the analysis of the precise mechanism underpinning NET1-mediated GC cell invasion revealed that NET1 is an activator of RhoA protein. The authors speculated that NET1 is a key player in the activation of RhoA and the subsequent migration and invasion of GC cells [61].

Another process observed during EMT is the degradation of ECM proteins that confer cells with invasive potential. Recent studies have suggested that ECM remodeling could be mediated by oxidative stress. Integrins, the cell surface adhesion molecules that link the ECM to the intracellular actin cytoskeleton, can undergo oxidative modification by ROS during the initiation of EMT [58]. Moreover, the elevated ROS levels could contribute to cancer metastasis by regulating the metalloproteinases (MMPs). MMPs have been identified as key enzymes in the EMT process as they are capable of degrading ECM components, specifically basement membrane, proteoglycan, fibronectin, and collagen [62]. Hung et al. have shown the link between mitochondrial dysfunction, and GC progression by enhancing migration through mitochondria-generated ROS mediated β5-integrin overexpression [63]. They found that the protein expression of β5-integrin is a key player in the ROS-induced cell migration of the SC-M1 cell line with mitochondrial dysfunction induced by specific inhibitors, oligomycin, and antimycin A [63]. More importantly, Kawahara et al. found that expression of β5-integrin is closely associated with invasive behavior in GC patients [64]. These results are of considerable importance for the identification of chemotherapeutic agents that modulate the cellular ROS content and may have the potential for clinical application in preventing or delaying GC metastasis.

The additional findings supporting the importance of ROS during EMT is that ROS could regulate the MMPs expression levels that are closely correlated with ECM stabilization. Cai et al. focused on the unique roles of 18β-glycyrrhetinic acid (18β-GA), a bioactive component of licorice root, in the GC metastasis process [62]. They have shown that 18β-GA significantly reduced invasion and migration potency by MMP-2 and MMP-9 activities suppression in SGC-7901 gastric cell line. In the same study, the authors revealed that 18β-GA inhibited ROS formation. The speculated mechanism of 18β-GA invasion and migration inhibition relates to ROS/PKC-α/ERK signaling pathway [62]. Protein kinase C-α (PKC isoenzyme α) is implicated in multiple pathways and controls the expression of genes relevant for cell cycle progression, tumorigenesis, and metastatic dissemination [65]. PKC-α can also be regulated by ROS to become involved in various cell signaling mediators, including the PKC/ERK pathway [62]. Mitogen-activated protein kinase 1 (ERK) is involved in the activity of MMPs, E-cadherin, and vimentin in various cancers [62]. Thus, inhibition of ROS formation by 18β-GA could inhibit PKC-α in mediating the phosphorylation of ERK. Inactive ERK cannot effectively regulate MMP-2 and MMP-9 that reduce an aggressive migratory phenotype of GC cell line. Similar results concerning the role of ROS in MMP-2 and MMP-9 regulation in GC BGC-823 cell line were presented by Qi et al. [66]. However, they have discovered the inhibition of cell MMP-2 and MMP-9 in response to salidroside, an active ingredient extracted from the *Rhodiola rosea* plant. Moreover, in the same study, the authors reported that salidroside treatment of GC cell enhanced the expression of E-cadherin and reduced the expression of N-cadherin. Thus, this study suggests an anti-tumor role of salidroside in EMT in GC cells [66]. The speculated molecular mechanism of salidroside inhibition of EMT relates to inhibition of ROS production and ROS-mediated Src-associated signaling pathways [66].

EMT is considered the initiating event for cancer metastasis and is characterized by the diminishment of cell junctions that are essential for maintaining epithelial integrity [58]. Cell junction proteins are repressed during EMT by transcription factors such as Snail, Slug, Twist, and zinc-finger E-box binding homeobox family (ZEB). Additionally, these key EMT-inducing transcription factors are regulated by the convergence of signaling pathways the oxidant-sensitive transcription factors HIF-1 and NF-κB [47,58]. HIF-1 is activated under low oxygen level conditions and works as a transcription factor inducing an adaptive response to hypoxia by regulating the expression of genes associated with angiogenesis, cell growth, metastasis, and glycolytic metabolism. Since hypoxia is a common element of the tumor environment, the HIF-1 activated pathways are critical for cancer cells survival, growth, and progression [47,67,68]. NF-κB is an oxidant-sensitive transcription factor that regulates the expression of a plethora of genes mostly involved in the cellular growth, apoptosis pathways, and antioxidant system [47,57,69]. The importance of NF-κB activity in cancer cells is the inhibition of ROS by activation of antioxidant enzymes. Thus, NF-κB acts against ROS accumulation in cells to maintain cellular oxygen radical homeostasis which, in turn, could attenuate chemotherapeutics activity [47,70]. Moreover, transcription of Snail is partly regulated by NF-κB and is a key modulator in metastasis [69]. Below, we will discuss the role of ROS regulation of signaling pathways HIF-1 and NF-κB implicated in EMT in GC. Qin et al. investigated the association of autophagy inhibition with the EMT promotion in GC cells. Autophagy is a dynamic metabolic process to maintain intracellular homeostasis and is used by cancer cells for survival. More importantly, the authors have shown that autophagy inhibition with the EMT promotion was dependent on the ROS-NF-κB-HIF-1α pathway [71]. The detailed studies revealed that autophagy inhibition increased the expression of mesenchymal markers such as N-cadherin, vimentin, Snail, Twist-1, and decreased expression of epithelial marker E-cadherin. Moreover, autophagy defect results in an increase in intracellular ROS level as well as mitochondrial ROS level [71]. The authors suggested that autophagy defect induced HIF-1α activation and EMT are based on ROS generation. They also found that increased ROS level caused by autophagy inhibition activates NF-κB a transcriptional regulator of the HIF-1α gene. Therefore, Qin et al. concluded that autophagy defect can induce EMT via the ROS-NF-κB-HIF-1α pathway [71]. Complementary to these results, Yang et al. found that hypoxia and activated HIF-1α may regulate Snail expression, leading to the induction of GC EMT-like CSCs. EMT-like CSCs are a subset of cells that display EMT phenotype and stem cell properties [72]. In this study, the authors have discovered that GC cell lines exposed to hypoxia showed enhanced expression of both HIF-1α and Snail initiating a cascade of events that leads to the changes characteristic of EMT, including decreased E-cadherin expression, increased vimentin expression, and enhanced invasion ability [72]. Thus, the authors speculated that the HIF-1α-Snail-EMT axis could potentially be a new target for therapeutic strategies for GC [72]. Besides, Farris et al. observed that the occurrence of EMT induces ROS neutralization by suppressing hydrogen peroxide formation and thus, protecting the cells against anoikis [73,74].

Taken together, EMT could be defined as a multifactorial and complex network that is regulated on the level of cytoskeleton remodelling, ECM, cell-cell junctions, and cell motility. We tried to demonstrate the evidence for an eminent role of ROS in gastric cancer EMT in the present section. As it was shown, ROS can influence the function of various key proteins such as RhoA, NET1, integrins, MMPs, epithelial, and mesenchymal markers involved in the EMT process. Moreover, ROS regulates signaling pathways HIF-1 and NF-κB, as well as their downstream targets Snail, Slug, Twist, and ZEB, which are implicated in EMT in GC. Therefore, it seems reasonable to put an effort into further research to find the key ROS response EMT predictor that could attenuate EMT progression.

## 5. Current Treatment Strategies for EMT Inhibition in Gastric Cancer

### 5.1. Polyphenols

Polyphenols are the molecules with at least one aromatic ring with one or more hydroxyl functional groups attached. These compounds are secondary metabolites of plants involved in the signal transduction, as well as defense against ultraviolet radiation or aggression by pathogens [75]. Foods and beverages of plant origins (e.g., fruits, vegetables, spices, nuts, wine, and tea) are the source of natural polyphenols [76,77]. Based on the chemical structures, natural polyphenols can be divided into five major classes flavonoids, phenolic acids, lignans, stilbenes, and other polyphenols, of which flavonoids are the most abundant [78].

The anticancer efficacy of polyphenols has mainly been attributed to their antioxidant properties [79,80]. They also display active participation in cancer pathways, in particular, signaling pathways, which are associated with cell survival, proliferation, differentiation, metastasis, angiogenesis, hormone activities, etc. [81,82,83]. In the case of GC, accumulating evidence from laboratory studies has supported the EMT reversal properties of polyphenols.

#### 5.1.1. Resveratrol

Among polyphenols, resveratrol has attracted researchers’ attention because of its cardioprotective and anticancer properties [84]. It is predominantly found in red wine, grapes, and berries [85]. In GC cells (AGS, BGC-823, and SGC-7901), resveratrol treatment (25 and 50 μM) arrested cancer cells in the G1 phase by dysregulation of cyclin D1, cyclin-dependent kinase (CDK4 and 6), p21 and p16, resulting in senescence instead of apoptosis. Similarly, resveratrol (40 mg/kg/day) inhibited GC development in nude mice. The inhibition effects of resveratrol on GC acts in a Sirt1-dependent manner [86]. At higher concentrations (50–200 μM), resveratrol induced DNA damage and apoptosis in human gastric adenocarcinoma cells via promoting the generation of ROS [87].

Doxorubicin is a chemotherapeutic drug that is primarily used against GC. However, long-term exposure to doxorubicin in GC patients leads to the development of drug resistance by induction of EMT [88]. Xu et al. study showed that resveratrol reverses doxorubicin resistance in GC by preventing EMT through controlling PTEN/Akt signaling pathways. A doxorubicin-resistance GC cell line was developed by using a doxorubicin concentration gradient method in SGC7901 cells. Resveratrol enabled SGC7901/DOX cells to regain doxorubicin sensitivity, mitigated the aggressive biological features, promoted cell apoptosis in vitro, and inhibited tumor growth in a nude mice xenograft model. This study verified that resveratrol suppressed Akt signaling pathway by upregulating PTEN. In addition, the combination of doxorubicin and resveratrol synergistically enhanced caspase-3 and reduced vimentin and Ki-67 [89]. It was demonstrated that the combination of doxorubicin with *Allium* species might be beneficial in exerting toxic effects on GC cell lines by the restoration of CDH1 and COX2 downregulation [90].

Another way through which resveratrol reverses EMT is by inhibiting the hedgehog (Hh) signaling pathway. Gli-1 is a key component of the Hh pathway and is also regarded as a marker of its abnormal activation [91]. Gli-1 can induce the expression of Snail, thus decreasing the expression of E-cadherin and increasing the expression of N-cadherin [92]. Resveratrol inhibited Gli-1 expression, and then downregulated Snail and N-cadherin expression, and upregulated E-cadherin expression in SGC7901 cells [93].

#### 5.1.2. Tangeretin

Tangeretin is a member of polymethoxyflavones mainly found in the peel of citrus fruits [94]. Studies have shown that tangeretin exhibited broad bioactivities including antioxidant, anti-inflammatory, antidiabetic, and neuroprotective effects [95,96,97,98]. It induces apoptosis in GC through the up-regulation of the *RARB* gene expression and activation of caspase-3, caspase-9, and PARP1 [99].

Xukui et al. investigated the ability of tangeretin to enhance radiosensitivity in radiation-induced EMT of GC cells. Tangeretin enhanced the radiosensitivity of SGC7901 cells and suppressed irradiation-induced EMT and metastasis both in vitro and in nude mice model, possibly due to the inactivation of the Notch-1 signaling transduction and the up-regulation of miR-410 [100]. miR-410 acts as a tumor suppressor by targeting the *MDM2* gene and inhibiting GC cell proliferation and metastasis [101].

#### 5.1.3. Luteolin

A Noth-1 signaling pathway is also affected by luteolin treatment. Luteolin is a flavone abundant in artichoke and several spices, including sage, thyme, and oregano [102]. In Zang et al. study, luteolin reversed EMT by inducing the expression of epithelial biomarker E-cadherin and downregulating the mesenchymal biomarkers N-cadherin, vimentin, and Snail. Luteolin also suppressed Noth-1 signaling in GC cells (NCI-N87, MKN28, Hs-746T). In an in vivo assay, luteolin suppressed tumor growth by inhibiting proliferation and inducing apoptosis [103].

#### 5.1.4. Genistein

Genistein is an abundant isoflavonoid contained in soy and soy products, it is also a major active component of hormonal supplements for menopausal women [104]. Genistein treatment (15 μM) suppressed the GC cell stem-like abilities such as self-renewal, drug resistance, and carcinogenicity; possibly due to down-regulation of stemness-related genes as well as drug resistance gene *ABCG2* [105].

Xiaozheng et al. evaluated the effect of genistein synthetic analogue-7-Difluoromethoxyl-5,4′-di-n-octyl genistein (DFOG) on GC stem-like cells. GC stem-like cells were obtained from the SGC-7901 cell line and possessed mesenchymal characteristics including migratory and invasion properties as well as high expression of N-cadherin. DFOG treatment reversed the EMT process and inhibited cell migration and invasion. At the molecular level, these effects were accompanied by the downregulation of forkhead box M1 (FoxM1) and suppression of Twist1 protein [106]. By inhibiting Twist1, DFOG acts synergistically on the reversion of EMT because Twist1 causes the upregulation of FoxM1 [107]. Downregulation of FoxM1 leads to the inhibition of EMT in GC cells [39].

### 5.2. Proton Pump Inhibitors

Proton pump inhibitors (PPIs), such as omeprazole, esomeprazole, and pantoprazole, have been widely used to treat a variety of acid-related disorders, including gastroesophageal reflux disease (GERD), peptic ulcer disease, as well as *H. pylori* infections [108]. PPIs exert their action through irreversibly inhibiting H+/K+-ATPase proton pumps in the gastric parietal cells, hence inhibiting gastric acid secretion [109]. Emerging data show that PPIs might function as a prospective anticancer agent. PPIs prevent intracellular proton extrusions in GC cells consequently reducing cancer cell survival under acidic conditions [110]. Gu et al. reported that rabeprazole reduces cell viability of human GC cells through inactivation of the ERK1/2 signaling pathway [111].

Wnt/β-catenin signaling pathway plays a crucial role in regulating EMT in GC [112]. Zhang et al. study showed that pantoprazole suppresses the invasiveness of the doxorubicin-resistant GC cell model (SGC7901/DOX) by targeting the EMT and Akt/GSK-3β/β-catenin signaling. An SGC7901/DOX cell line was developed by using increasingly higher concentrations of doxorubicin. SGC7901/DOX cells underwent EMT and displayed mesenchymal phenotype as well as hyper-activated Wnt/β-catenin signaling. Pantoprazole treatment inhibited the Wnt/β-catenin signaling in SGC7901/ADR cells and reduced the expression of Tcf4, but not c-myc and cyclin D1, which are all the well-known Wnt/β-catenin downstream target genes [113].

In a study by Feng et al., pantoprazole treatment applied to GC stem-like cells (SGC-7901 and HGC-27 lines) reduced the expression of stem cells’ markers (CD44, CD24, ABCG2, EpCAM, Lgr5), decreased proliferation, and enhanced 5-FU chemosensitivity via suppression of EMT and β-catenin signaling [114].

### 5.3. Terpenes

#### 5.3.1. Ursolic Acid

Ursolic acid (UA)-is a natural triterpene isolated from, among others, rosemary leaves, marjoram, lavender, thyme, and organum, as well as some fruits and flowers [115]. UA shows neuroprotective [116], antioxidant [117], hepatoprotective [118], anti-carcinogenic [119], antidiabetic [120], anti-inflammatory [121], anti-obesity [122], cardioprotective [123], anti-skeletal muscle wasting [124], and thermogenic effects [125], and by mediating the pharmacological processes and modulating signaling pathways, it prevents the development of chronic diseases [126,127]. Several mechanisms have been proposed that may explain the beneficial pharmacological effects by Seo et al.: UA is involved in the regulation of the atrophic and metabolic signaling in skeletal muscle, inflammation and antioxidant levels in the brain, NF-κB, and apoptotic signaling in cancer cells, metabolic signaling and oxidant levels in the liver, insulin signaling in tissues, and the expression of markers of heart damage in the heart [115]. Numerous in vitro and in vivo studies have confirmed the anti-cancer properties of this compound through the following mechanisms: Promoting autophagy [128,129], modulating apoptosis [130], inhibiting oncogenesis [131], and proliferation of cancer cells [132], and preventing the cell cycle arrest [19]. There are attempts to apply the UA in inhibiting EMT, especially in GC. Li et al. investigated the influence of UA on the expression of key proteins in the Axl/NF-κB pathway [133,134]. UA, depending on the dose, induces apoptosis as well as inhibits cell migration and proliferation. In mice, the GC xenographic model showed a decrease in p-Axl and p-IKK; also, a solution of UA at a concentration of 25 mol/L UA caused a decrease in the levels of p-Axl, p-IKK α/β, and p-NF-κB B in BGC-823 cells. At the same time, the authors showed that the use of UA at a concentration of 50 µmol/L caused significant damage to MGC-803 and AGS cells, excluding them from further studies. This points to the need for more research to find a safe, effective dose of UA. The results of the research by Li et al. are consistent with the previous studies [135,136].

#### 5.3.2. Astragaloside IV

Astragalus saponine IV (Astragaloside IV) (AG]) is a lanolin alcohol-shaped tetracyclic triterpenoid saponin isolated from *Mongolia Astragalus* [137]. AG shows neuroprotective [138,139,140], heart-protective [141,142], and hepatoprotective [143,144] properties; it also improves endocrine system [145,146], enables proper endothelial function [147,148], regulates collagen decomposition and synthesis [148,149,150,151], and protects the hematopoietic system [152]; it can also be used to treat cancer by boosting immunity [153]. Qi et al. observed a decrease in the expression of Vav 3.1 oncogenes by AG, which resulted in an anti-proliferative effect on HepG2 cells [154]. In the HepG2/glucosylceramide (GSM) synthase of cells, the possibility of reducing the expression of GEN GCS in cells by AG has also been suggested, which may reverse a multi-drug resistance in HepG2/glucosylceramide (GSM) synthase of cells [155]. AG also reduced Akt.3 phosphorylation in the human breast cancer MDA-MB-231 cell line as shown by an effect on their proliferation resistance [156]. Li et al. showed that in rats AG stimulates the expression of an immune costimulatory factor on the surface of dendritic cells and the main molecule of the histocompatibility complex. In addition, to develop the antenna presentation and induce T cell responses, there is an increase in the secretion of IL-2 and IL-6, the development of the antenna presentation, and the induction of T cell responses [152]. In traditional Chinese medicine, treatment of gastrointestinal tumors by toning the Qi and activating the blood circulation provides a major immunological basis [157]. There are attempts to use AG in inhibiting EMT, especially in GC. Zhu and Wen showed that the invasion, migration, and viability of GC cell lines by AG at concentrations of 5, 10, 20, or 40 μg/m were inhibited [158]. AG was observed to reverse the E-cadherin to N-cadherin conversion and the expression of vimentin genes and serving genes metastatic, induced by TGF-β1. The activation of PI3K/Akt/NF-κB induced by TGF-β1 was also inhibited by AG. This is indicative of an inhibition of TGF-β1-induced EMT by inhibition of the PI3K/Akt/NF-κB pathway in the GC cells by AG.

### 5.4. Plant Extracts

#### 5.4.1. *Celastrus orbiculatus*

*Celastrus orbiculatus* (COE) is a plant with anti-inflammatory properties that has been used in traditional Chinese medicine for centuries. It has been used in rheumatoid arthritis, insomnia, and contusion [159]. In addition, it has properties analgesic [159], hypnotic [159], and anti-tumor [160,161,162,163] properties. Zhou et al. demonstrated that β-dihydroagarofuran sesquiterpenes from COE exhibited antiproliferative activity, directed against human colon cancer HCT-116 cells, human acute promyelocytic leukemia HL-60 cells, and human leukemic K562 cells [160]. Qian et al. demonstrated the existence of a synergistic inhibition of hepatocellular carcinoma growth through low mTOR expression and COE-induced cell apoptosis in HepG2/mTOR-in vitro [161]. Additionally, COE lowered the level of Bcl-xL and Bcl-2 expression, while increasing the level of Bax and caspase-3 in vivo [161]. Qian et al. in another study also found that COE inhibits the expression of VRGF at the mRNA and protein level and inhibits proliferation while inducing apoptosis in Hepa1-6 cells. Moreover, in in vivo studies, COE reduced tumor angiogenesis as well as tumor volume and mass. [162]. In still other studies, Qian et al. demonstrated a synergistic effect of COE on masapine expression, thereby inducing apoptosis while simultaneously inhibiting invasion and migration in GC cells MGC803. This is done due to the inhibition of MAPK and PI3K/Akt/mTOR signaling pathways and the regulation of proteins related to apoptosis [163]. There are attempts to use UA in inhibiting COE, especially in GC. Zhu et al. showed that COE reduces the expression of N-cadherin, vimentin, MMP-2, and MMP-9 while increasing the expression of E-cadherin in a dose-dependent manner [164]. Thus, it has been shown that by suppressing the PHB/c-Raf/ERK signaling pathway, COE inhibits the invasion and migration of GC cells MGC-803 [164]. In other studies, Zhu et al. demonstrated inhibitory effects of COE on EMT and the NF-κB/Snail signaling pathway of human GC SGC-7901, resulting in growth inhibition and anti-metastasis in nude mice models [165]. However, the authors note that further studies are needed to confirm the molecular mechanisms of COE action in other types of cancer and to confirm the metabolism and pharmacokinetics of COE [165]. Zhu et al. demonstrated that COE increased the expression of E-cadherin while decreasing the expression of N-cadherin and vimentin in rats [166]. It is worth adding that Zheng et al. related E-cadherin, N-cadherin, and vimentin with the expression of Lgr5 [167]. This indicates that COE can inhibit Lgr5 expression and proliferation of Lgr5þ cells [166]. These findings allow us to speculate that COE may reverse precancerous lesions of GC by inhibiting Lgr5 and EMT in gastric epithelial cells [166].

#### 5.4.2. *Trametes robiniophila*

*Trametes robiniophila* (TR)-is a mushroom from traditional Chinese medicine. The proteoglycan, consisting of polysaccharides, amino acids, and water, is the main active ingredient of TR [168]. A satisfactory clinical effect of TR has been demonstrated on nephrosis [169], colitis [170], tuberous sclerosis, [171], and cancer [172]. TR inhibited proliferation and metastasis in the tuberous sclerosis complex by attenuating JAK2/STAT3 and MAPK signaling in Tsc1- or Tsc2-null mouse embryonic fibroblasts (MEFs) [171]. Bai et al. demonstrated that in rats, TR inhibits mesanium DNA synthesis and inhibits proliferation, which is stimulated by the platelet growth activity of BB in mesangial proliferative glomerulonephritis [169]. TR has strong anti-tumor properties against hepatocellular carcinoma (HCC) [173,174], as well as breast [175,176], ovarian [177], and gastrointestinal cancers [178]. This confirms the effectiveness of TR in adventitious cancer therapy. Pan et al. mentioned the mechanisms responsible for the anti-tumor activity, such as inhibition of cancer cell proliferation through the cell cycle arrest, enhancement of iκBα expression to inhibit NF-κB-mediated signaling pathway, inhibition of NF-κB–estrogen receptor (eR) pathway, inhibition of Pi3K–Akt pathway, inhibition of Yes-associated protein 1 (YAP1) expression, suppression of CSCs, induction of cancer cell death through stimulation of autophagy, induction of apoptosis, inhibition of tumor-induced angiogenesis, or suppression of cancer metastasis [168]. Ji et al. showed that TR induces apoptosis in the human GC MKN-45 cell line through mitochondrial and the member receptor signaling pathways. TR, by reducing the expression of MMP-2 and MMP-9, inhibits the ability of GC cells to metastasize [179]. TR by reducing the expression of PI3K inhibits the expression of AKT, p-AKT, PTEN, and p-PTEN. Inhibition of p-AKT is mediated by reducing the expression of the activated form of caspase-9. The authors suggest that TR, by inhibiting Pi3K and PDK1 expression, inhibits AKT phosphorylation. A decrease in AKT phosphorylation leads to a decrease in the expression of pro-caspase-9 and Bcl-2. TR by modulating the PI3K/AKT pathway by inhibiting PI3K expression induces apoptosis of human GC cells [179]. Zhenga et al. demonstrated that TR polysaccharides inhibit invasion and migration of hepatocellular carcinoma cells by blocking AEG-1 signaling and restoring EMT in MHCC97-H cells [180]. Xu et al. showed that TR caused decreased expression of N-cadherin, vimentin, and increased expression of E-cadherin. In addition, TR has been shown to inhibit metastasis of GC cells by regulating EMT. TR, by reducing the Twist response, can reverse EMT in GC cells SGC7901 both in vitro and in vivo. The authors also suggest that TR may regulate Twist through mediated indirect expression PI3K/AKT signaling [181].

#### 5.4.3. *Poria cocos*

*Poria cocos* is a saprophytic mushroom that grows in various Pinus species. Due to its diuretic, sedative, and tonic properties, it is widely used in traditional Chinese and Japanese medicine [182]. Moreover, it has the antinephritic [183], antiviral [184], antiparasite [185], antiemetic [186], antioxidant [187], anti-inflammatory [188,189,190,191], immunomodulatory [192,193,194,195,196], and antihyperglycemic [197,198,199], as well as anti-cancer properties. Dehydroeburic acid and dehydrotramethenonic acid stopped the growth of human GC cells in the G1 phase of the cell cycle, thus preventing its development [182]. Yance and Sagar linked the anti-tumor effects of *Poria cocos* with the ability to reduce NF-κB expression, resulting in inhibition of angiogenesis [200]. Wang et al. obtained sulphate and carboxymethylated β-d-glucan derivatives isolated from *Poria cocos*, which showed significant antitumor activity, among others, on GC cells MKN-45 and SGC-7901 [201]. Chen et al. showed clinical evidence that the combination of *Poria Covos* and chemotherapy can alleviate the side effects associated with chemotherapy and may improve the tumor response rate [202]. *Poria cocos* has been shown to increase the sensitivity of cancer cells to chemotherapy and has almost no side effects [203].

Oxaliplatin (oxalato (trans-l-1,2-diaminocyclohexane) platinum), is distinguished from cisplatin by the presence of diaminocyclohexane groups instead of the main groups [204]. Alcindor and Beauger mention the following mechanisms of action of oxaliplatin: DNA damage by cytotoxic action, tumor cell apoptosis by stopping DNA and RNA synthesis, as well as inducing immunological reactions and changes in DNA and synergism of action with other cytotoxic drugs [204]. It is used in the treatment of the gastrointestinal or gastrointestinal cancer system [203]. Wang et al. in the in vitro studies showed that *Poria cocos* in combination with oxaliplatin reduces mRNA and the expression of N-cadherin and vimentin while increasing mRNA and protein expression of E-cadherin, which results in reduced migration and invasive capacity of GC cells SGC7901 [203]. These results are relevant to other articles [205,206]. In in vivo studies in nude mice, the same authors showed that there was a decrease in the expression of Snail, Twist, vimentin, and N-cadherin with a simultaneous increase in E-cadherin expression in the group of *Poria cocos* and oxaliplatin treatment [203]. This demonstrates the possible inhibition of the EMT process of GC by the combination of *Poria cocos* and oxaliplatin.

### 5.5. Inorganic Compounds

#### 5.5.1. Arsenic Derivatives

Arsenic derivatives have been used in medicine for over 2400 years [207]. The dose-dependent nature of this compound was known to be twofold-medicinal or poisonous [208]. Litzow lists the following mechanisms of apoptosis induction by arsenic oxide-glutathione depletion, induction of intracellular ROS, activation of kinases (e.g., c-jun N-terminal kinase), downregulation of telomerases wt-1 and BCL-2, inhibition of NF-κB, caspase activation, inhibition of p-glycoprotein, and potentiation of tubulin polymerization [209]. Ma et al. demonstrated that As2O3 induces apoptosis of GC cells, while not causing any serious side effects [210]. Furthermore, As2O3 was reported to induce apoptosis by blocking BGC-823 GC cells in the G0/G1 phase [211]. Gu et al. demonstrated inhibition of the proliferation of MKN45 and SGC7901 by As2O3 through the induction of apoptosis [212]. Kim et al. showed that As2O3 inhibits EMT and cell invasion in AGS cells by modulating Snail1/E-cadherin expression and increasing SHP-1 expression to dephosphorylate JAK2/STAT3 [213]. It is worth mentioning that STAT3 plays a key role in carcinogenesis, invasion, and modulation of the GC microenvironment. Its constitutive activation promotes invasion, angiogenesis, and proliferation of cells and through activation of D1, VEGF-1, Bcl-xL, survivin, and MMP-9, it inhibits apoptosis [214,215]. It has been reported that in normal gastric tissues, SHP-1 mRNA expression was higher, and the reverse was seen in GC, so stimulation of SHP-1 expression may inhibit and dephosphorylate STAT3 in GC [213].

#### 5.5.2. Cisplatin

Cisplatin (*cis*-diamminedichloroplatinum (II)) has been proven to fight sarcomas, tumors of soft tissue, bones, muscles, and blood vessels. As new therapeutic strategies, cisplatin is used along with other anti-cancer drugs due to increasing drug resistance and the problem of harmful side effects. Florea and Büsselberg list the following mechanisms of the cellular activity of platinum compounds: Induction of ROS, cytotoxicity, extrinsic apoptosis, and intrinsic apoptosis [216]. One of the problems with the use of cisplatin is the phenomenon of drug resistance. Drug resistance is the failure of cancer cells to respond to treatment with anti-cancer drugs. There are two types of resistance: Intrinsic, when the drug becomes ineffective from the start of treatment, and acquired when the drug becomes ineffective over time [216]. Drug resistance is responsible for the failure of chemotherapy in cancer patients [217,218]. In the case of cisplatin, there is a risk of acquired drug resistance, which results in serious complications of therapy, because a too-high dose may result in severe multi-organ toxicity [217]. Several mechanisms responsible for cisplatin resistance in cancer cells are listed, including drug inactivation, accumulation and/or increased drug efflux, changes in target drug, and increased nucleotide excision repair activity that processes drug-induced damage, with reduced mismatch repair activity and avoidance of apoptosis [217,219,220]. Ashrafizadeh et al. collected the articles available so far that describe the induction of cisplatin resistance by the EMT mechanism [221].

### 5.6. Monoclonal Antibodies

Monoclonal antibodies (MA) are artificially made proteins that have to act as human antibodies when introduced to the immune system. A classification of MA distinguishes four major classes depending on the origin and process of antibody formation: Murine, chimeric, humanized, and human MA. The uniqueness of MA is mainly due to their high affinity to the antigens numerously expressed on the surface of cancer cells. MA are approved in the treatment of numerous hematological malignancies as well as solid tumors including brain cancer, breast cancer, colorectal cancer, prostate cancer, melanoma, lung cancer, and GC.

Trastuzumab is HER-2 targeting antibody useful in GC patients with HER2 overexpression. It was demonstrated that the combination of trastuzumab with fluorouracil and cisplatin provides promising results in the case of HER2-positive advanced GC. Besides, ramucirumab (which is also an antiangiogenic agent) also shows its efficacy in metastatic GC [222]. Except for ramucirumab, bevacizumab, another anti-VEGF antibody, might be applied to treat GC by inhibiting angiogenesis. To prevent pathological angiogenesis in the GC microenvironment, anti-EGFR agents such as cetuximab, panitumumab, or matuzumab can also be used [223].

It was shown that EMT itself stimulates mechanisms that induce resistance to lapatinib or trastuzumab, which are proven to be effective in GC therapy. Moreover, GC cells resistant to the above-mentioned agents usually show the upregulation of the EMT-related gene signatures, which might hinder GC treatment [224].

### 5.7. Other Treatment Strategies

Except for the above-described treatment agents included in particular groups, several therapies that cannot be included there are described in this section. As previously mentioned, the induction of EMT in the gastric microenvironment is usually associated with the progressive resistance to many therapeutic agents, which impedes GC treatment. Thus, numerous new agents are continually investigated alone or combined to improve the clinical outcome of patients with GC.

Salinomycin is an antibiotic extracted from *Streptomyces albus* which recently gathered researchers’ interest since it induces apoptosis of cancer cells in a vast number of human tumors. Salinomycin-induced cellular death is facilitated by the caspase-3 activation, accumulation of ROS, and depolarization of the mitochondrial membranes [225]. Mao et al. demonstrated that salinomycin might be a potential EMT inhibitor since, in his study, the administration of salinomycin in SGC7901/CDDP cells resulted in a significant increase in epithelial markers (E-cadherin and ZO-1) and a decrease in the mesenchymal markers (N-cadherin, vimentin, Twist, ZEB-1) [226].

Diallyl disulfide (DADS) is a sulfur compound derived from garlic presenting a wide spectrum of anti-tumor properties including the inhibition of angiogenesis, invasion, metastasis, cellular growth, and differentiation; it also facilitates the apoptosis of the cancer cells. DADS was shown to downregulate the Rac1-Pak1/Rock1-LIM kinase-1 (LIMK1) pathway in gastric MGC803 cells decreasing the LIMK1 levels [227]. DADS upregulates microRNAs (miR-200b and miR-22 in particular) inhibiting the Wnt-1 pathway, which is associated with further pro-apoptotic properties of this compound. By downregulating LIMK1 levels, DADS is considered to be a potential suppressor of EMT in the gastric microenvironment. Additionally, DADS inhibits the TGF-β1 expression, preventing the induction and further progression of EMT [228]. Similar results regarding TGF-β1 inhibition were obtained while applying sauchinone in GC cells [229]. Sauchinone is an active compound isolated from *Saururus chinensis* that possesses hepatoprotective, anti-inflammatory, as well as anti-tumor properties. It is now considered as a potential therapeutic agent targeting EMT in GC patients.

Eribulin is a methanesulfonate salt obtained during a reaction of eribulin with methanesulfonic acid. Eribulin induces cellular apoptosis by blocking mitosis in the G2/M phase. Eribulin was described as a factor that might inhibit or even reverse EMT in several cancers including breast cancer [230]. Kurata et al. demonstrated that eribulin mesylate (eribulin) might also be a potential therapeutic agent applied for GC treatment [231]. In Kurata’s study, it was demonstrated that eribulin administration increases E-cadherin expression while decreasing α-SMA and vimentin levels; besides, the researchers showed synergy between eribulin and 5-fluorouracil. Such a combination is considered to be a potential therapy preventing peritoneal dissemination of GC.

Another treatment agent that might be applied in GC patients is metformin. Metformin is an antidiabetic drug primarily used in diabetes type II, however its application spectrum has been quite recently broadened due to its anti-tumor properties and now there are attempts to use this drug in several cancers such as lung, prostate, breast, and stomach cancers, or even glioblastoma [232,233,234]. It was demonstrated that GC progression might be inhibited by metformin by targeting the HIF1α/PKM2 signaling pathway [235]. Besides, metformin seems to be beneficial in decreasing cellular migration and invasion properties. It was also shown that metformin (at the range of 2.5–50 mM) might inhibit EMT progression by increasing E-cadherin levels and the same time decreasing the levels of the mesenchymal markers–vimentin and β-catenin [236].

Dextran sulfate (DS) is a macromolecule Dextran, considered as another potential treatment agent targeting EMT in GC patients. Generally, DS impairs cellular adhesion, gene expression, and cell cycle progression. In GC cells, DS might affect angiogenesis and cellular adhesion by targeting VEGF and integrin β1, respectively [237]. Xu et al. demonstrated DS relevance in GC treatment by showing that DS increases E-cadherin while decreasing N-cadherin and Twist levels, thus contributing to the reversal of EMT in the gastric microenvironment [238].

Crocin is a carotenoid chemical compound primarily found in saffron that exhibits a wide spectrum of beneficial properties including antioxidant, anti-inflammatory, anti-tumor, or even antidepressant ones. Zhou et al. demonstrated that crocin might inhibit EMT at the same time reducing the migration and invasion properties of GC cells in AGS and HGC-27 cell lines by modulating miR-320/KLF5/HIF-1α signaling. [239].

## 6. Conclusions

EMT constitutes one of the most crucial molecular targets enabling developing new pharmacological agents that aim to prevent the progression of GC. At the current state of knowledge, targeting EMT is considered to be an effective strategy for GC treatment. It was demonstrated that several agents that target EMT not only inhibit but might even reverse this process. The introduction of EMT inhibitors as an addition to standard GC treatment therapies might be associated with better clinical outcome, improving the current strategies of GC treatment. Moreover, targeting the EMT-inducing transcription factors seems to be crucial in the prevention of chemoresistance in GC. Research that aims to find therapeutic agents targeting EMT is crucial in terms of GC treatment since EMT itself induces resistance to numerous anti-agents inducing the risk of greater morbidity and mortality rates of patients.

## Abbreviations

18β-GA	18β-glycyrrhetinic acid
AG	Astragalus saponine IV
ATO	arsenic trioxide
CAT	catalase
COE	*Celastrus orbiculatus*
CSC	cancer stem cell
DADS	diallyl disulfide
DFOG	difluoromethoxyl-5,4′-di-n-octyl genistein
DS	dextran sulfate
EBV	*Epstein-Barr Virus*
ECM	extracellular matrix
EMT	epithelial-mesenchymal transition
ERK	mitogen activated protein kinase 1
ETS	electron transport system
FAK	focal adhesion kinase
FoxM1	forkhead box M1
GC	gastric cancer
GERD	gastroesophageal reflux disease
GPxs	glutathione peroxidases
*H. pylori*	*Helicobacter pylori*
HCC	hepatocellular carcinoma
HIF-1α	hypoxia-inducible factor
LIMK1	LIM kinase-1
MA	monocloncal antibodies
MEFs	mouse embryonic fibroblasts
MET	mesenchymal-epithelial transition
MMPs	metalloproteinases
NET1	neuroepithelial cell transforming gene 1
NF	κB-nuclear factor kappa B
NSAIDs	non-steroidal anti-inflammatory drugs
PDCD4	programmed death cell protein 4
PKC isoenzyme α	protein kinase C-α
PPIs	proton pump inhibitors
RhoA	ras homolog gene family member A
ROS	reactive oxygen species
SESN	sestrins
SOD	superoxide dismutase
Src	proto-oncogene tyrosine-protein kinase Src
TR	*Trametes robiniophila*
Trx	hioredoxin
YAP1	Yes-associated protein 1
ZEB	zinc-finger E-box binding homeobox family
ZO-1	zona-occludens 1
